# Timing and morbidity of loop ileostomy closure after rectal cancer resection: a prospective observational multicentre snapshot study from Multidisciplinary Italian Study group for STOmas (MISSTO)

**DOI:** 10.1007/s00384-025-04827-8

**Published:** 2025-02-18

**Authors:** Gianluca Rizzo, Francesco Ferrara, Dario Parini, Francesco Pata, Cristiana Forni, Gabriele Anania, Alessandro Anastasi, Gian Luca Baiocchi, Luigi Boccia, Diletta Cassini, Marco Catarci, Giovanni Cestaro, Nicola Cillara, Francesco Cobellis, Raffaele De Luca, Paola De Nardi, Simona Deidda, Daniele Delogu, Massimo Fedi, Maria Carmela Giuffrida, Ugo Grossi, Harmony Impellizzeri, Antonio Langone, Andrea Lauretta, Francesca Lo Celso, Anna Maffioli, Michele Manigrasso, Chiara Marafante, Luigi Marano, Peter Marinello, Paolo Massucco, David Merlini, Luca Morelli, Marta Mozzon, Donato Paolo Pafundi, Gianluca Pellino, Roberto Peltrini, Adolfo Petrina, Diego Piazza, Claudio Rabuini, Aridai Resendiz, Beatrice Salmaso, Mauro Santarelli, Giuseppe Sena, Leandro Siragusa, Nicolò Tamini, Vincenzo Tondolo, Roberta Tutino, Alberto Vannelli, Marco Veltri, Leonardo Vincenti, Andrea Bondurri, MISSTO Snapshot Study Collaborative Group, Francesco Bagolini, Matteo Chiozza, Sabrina Pedon, Giuseppe Canonico, Carmela Di Martino, Elvira Adinolfi, Manuela Mastronardi, Massimo Petrella, Guido Mantovani, Annalisa Pascariello, Gianandrea Baldazzi, Marta Spalluto, Marco Della Sanità, Maria Sole Mattei, Michele Benedetti, Leonardo Montemurro, Corrado Bottini, Gianluca Grillone, Antonello Deserra, Alessandro Cannavera, Luigi Cobellis, Roberto Scola, Francesca Savastano, Gabriele Carbone, Francesco Denti, Luigi Zorcolo, Angelo Restivo, Luca Ippolito, Fabrizio Scognamillo, Antonio Giulio Marrosu, Sandro Giannessi, Virna Robustelli, Marco Stella, Enrico Gelarda, Danilo Donati, Diego Sasia, Marco Piccino, Alberto Brun Peressut, Rino Baldan, Creciun Mihail, Alessandro Vitali, Gianluigi Moretto, Raffaele Galleano, Omar Ghazouani, Sara Pollesel, Claudio Belluco, Nicolò De Manzini, Fabio Porcelli, Alice Gabrieli, Andrea Micalef, Gloria Zaffaroni, Marco Milone, Giovanni Domenico De Palma, Sara Vertaldi, Ana Lavinia Apostu, Simone Lorenzo Birolo, Mauro Garino, Franco Roviello, Daniele Marrelli, Ludovico Carbone, Giacomo Bertelli, Antonio Frena, Federica Gonella, Marco Palisi, Federico Marin, Gregorio Di Franco, Niccolò Furbetta, Annalisa Comandatore, Cristina Folliero, Luca Amodio, Francesco Menegon Tasselli, Marco D’Ambrosio, Francesco Selvaggi, Biancamaria Iacone, Umberto Bracale, Roberto Ciaccarini, Michela Boncompagni, Davide Mascali, Caterina Piazza, Enrico Falzone, Rossella Reddavid, Maurizio Degiuli, Maurizio De Luca, Diego Visconti, Alice Ferguglia, Chiara Piceni, Giorgio Ammerata, Giuseppe Sica, Andrea Martina Guida, Bruno Sensi, Lorenzo Ripamonti, Giulia De Carlo, Paolina Venturelli, Gianfranco Cocorullo, Ada Della Valle, Andrea Romanzi, Maria Milanesi, Giovanni Tomasicchio, Nicola Paradiso, Ilaria Verriello

**Affiliations:** 1 Unit of Digestive and Colorectal Surgery, Ospedale Isola Tiberina Gemelli Isola, Catholic University of the Sacred Heart, Rome, Italy; 2 Department of Precision Medicine in Medical, Surgical and Critical Care (Me.Pre.C.C.), Unit of General and Oncologic Surgery, “Paolo Giaccone” Hospital, University of Palermo, Via Alfonso Giordano, 90127 Palermo, Italy; 3 Unit of General Surgery, Santa Maria Della Misericordia Hospital, Rovigo, Italy; 4 Department of Pharmacy, Health and Nutritional Sciences, University of Calabria, Cosenza, Italy; 5 Nursing and Allied Profession Research Unit, IRCCS Istituto Ortopedico Rizzoli, Bologna, Italy; 6 Unit of General Surgery 1, Arcispedale Sant’Anna, Ferrara, Italy; 7 Unit of General Surgery, San Giovanni Di Dio Hospital, Firenze, Italy; 8 Unit of General Surgery, ASST Cremona, Cremona, Italy; 9 Unit of General and Minimally Invasive Surgery, “Carlo Poma” Hospital, ASST Mantova, Mantova, Italy; 10 Unit of General Surgery, Legnano Hospital, Legnano, Italy; 11 Unit of General Surgery, Sandro Pertini Hospital, Rome, Italy; 12 Unit of General Surgery, San Antonio Abate Hospital, Gallarate, Italy; 13 Unit of General Surgery, Santissima Trinità Hospital, Cagliari, Italy; 14 Unit of General Surgery, Casa Di Cura “Prof. Dott. Luigi Cobellis”, Vallo Della Lucania, Italy; 15 Department of Surgical Oncology, IRCCS Istituto Tumori “Giovanni Paolo II”, Bari, Italy; 16 Unit of Gastrointestinal Surgery, San Raffaele Scientific Institute, Milan, Italy; 17 Unit of Coloproctology, Cagliari University Hospital, Cagliari, Italy; 18 Unit of Surgical Pathology, Sassari University Hospital, Sassari, Italy; 19 Unit of General Surgery, San Jacopo Hospital, Pistoia, Italy; 20 Unit of General Surgery, Cardinal Massaia Hospital, Asti, Italy; 21 DiSCOG Department, Unit of General Surgery 2, Treviso Regional Hospital, University of Padova, Padua, Italy; 22 Unit of General Surgery, Pederzoli Hospital, Peschiera Del Garda, Italy; 23 Unit of General and Oncologic Surgery, S. Paolo Hospital, Savona, Italy; 24 Unit of Oncologic Surgery for Sarcomas, Rare and Multi-Visceral Tumors, CRO IRCCS Aviano, Aviano, Italy; 25 Unit of General Surgery, Cattinara Hospital, Trieste, Italy; 26 Unit of General Surgery 1, Luigi Sacco University Hospital, ASST FBF-Sacco, Milan, Italy; 27 Unit of Endoscopic Surgery, Federico II University Hospital, Napoli, Italy; 28 Unit of General Surgery, Ospedale Degli Infermi, Rivoli, Italy; 29 Unit of Surgical Oncology, Le Scotte University Hospital, University of Siena, Siena, Italy; 30 Unit of General Surgery, Central Hospital, Bolzano, Italy; 31 Unit of General and Oncologic Surgery, AO Ordine Mauriziano, Torino, Italy; 32 Unit of General Surgery, Garbagnate Hospital, ASST Rhodense, Garbagnate Milanese, Italy; 33 Unit of General Surgery, Pisa University Hospital, Pisa, Italy; 34 Unit of General Surgery, ASUFC Udine Hospital, Udine, Italy; 35 Unit of General Surgery 2, Gemelli IRCCS University Hospital, Rome, Italy; 36 Unit of Colorectal Surgery, Primo Policlinico, Luigi Vanvitelli University of Campania, Napoli, Italy; 37 Unit of General and Oncologic Surgery, Federico II University Hospital, Napoli, Italy; 38 Unit of General Surgery, Perugia University Hospital, Perugia, Italy; 39 Unit of General and Oncologic Surgery, ARNAS Garibaldi, Catania, Italy; 40 Unit of General Surgery, Principe Di Piemonte Hospital, Senigallia, Italy; 41 Unit of General Surgery, San Luigi Gonzaga Hospital, Torino, Italy; 42 Unit of General and Emergency Surgery, AOU Città Della Salute E Della Scienza, Torino, Italy; 43 Dipartimento Specialità Chirurgiche, Pugliese-Ciaccio Hospital, Catanzaro, Italy; 44 UOSD Chirurgia Generale E Dell’apparato Digerente, Tor Vergata University Hospital, Rome, Italy; 45 Unit of General Surgery, San Gerardo Hospital, Monza, Italy; 46 Unit of General Surgery, Valduce Hospital, Como, Italy; 47 Unit of General Surgery, Azienda Ospedaliero Universitaria Consorziale Policlinico, Bari, Italy

**Keywords:** Ileostomy, Colorectal cancer, Surgery, Stoma, Ostomy

## Abstract

**Purpose:**

Time to closure and morbidity are significant issues associated with ileostomy reversal after rectal cancer resection. This study aimed to investigate the rate, time, and morbidity associated with ileostomy closure procedure.

**Methods:**

Between February and December 2022, patients who underwent protective ileostomy after rectal cancer surgery across 45 Italian surgical centres were prospectively included. Data on ileostomy closure times, surgical methods, and complications were collected and analyzed. Both univariate and multivariate statistical tests were employed to assess stoma closure rates and the occurrence of post-operative complications.

**Results:**

A total of 287 patients participated in the study. Ileostomy closure was achieved in 241 patients, yielding overall and 6-month closure rates of 84% and 62%, respectively. The median time for ileostomy closure was 146 days. Direct sutures were used to close approximately 70% of skin defects, while purse-string sutures were applied in around 20%. The overall morbidity rate was 17%, with complications including skin suture dehiscence (7%), small bowel obstruction (6%), and anastomotic leakage (2%). Multivariate analysis revealed that an American Society of Anesthesiologists (ASA) score > 2 (*p* = 0.028), advanced age (*p* = 0.048), and previous stoma complications (*p* = 0.048) were independently linked to failure of stoma closure; hypertension (*p* = 0.036) was found to be a significant independent risk factor for post-operative complications.

**Conclusion:**

This study demonstrated that a delay and a significant no-closure rate exist in ileostomy reversal after rectal cancer surgery. Post-operative complications remain high but can be prevented with adequate pre-operative assessment and post-operative care.

## Introduction

Anastomotic leakage (AL) is the primary issue following anterior resection with total mesorectal excision [1]. The incidence of AL ranges from 1.2 to 27%, and its occurrence has a significant impact on both short-term post-operative recovery and long-term functional and oncological outcomes [2–5]. Several studies have demonstrated the role of diverting ileostomy in reducing the clinical effects of AL in terms of post-operative mortality and major post-operative morbidities [6, 7]. However, ileostomy itself carries a notable risk of stoma-specific post-operative complications, which can negatively affect the stoma patients’ quality of life. In our recently published prospective multicentre snapshot study of 287 patients with diverting ileostomy after rectal cancer resection (enrolled at 1 year from 45 Italian colorectal surgery centres), the short- and long-term post-operative stoma complication rates were 33.8% and 29.6%, respectively, which were significantly higher in centres without a stoma nurse [8]. Additionally, stoma reversal seems to be associated with high post-operative morbidity rates of > 15% [9, 10]. Moreover, the prolonged presence of a temporary stoma, often performed to protect an ultra-low or at-risk colorectal anastomosis, is related to two other problems: first, the risk of a temporary stoma becoming definitive if an AL occurs, especially in frail patients [11, 12]; second, the long time to stoma closure, which may depend on several factors, such as the necessity of adjuvant therapy or hospital organization problems to rapidly plan the stoma closure procedure [11, 12]. In 2019 and 2021, guidelines about the surgical and nursing management of enteral stomas in adults were published by our group, underscoring the importance of a multidisciplinary approach to manage intestinal ostomies for optimal post-operative results [13, 14].

The primary aim of this study was to report the rate and timing of stoma closure, identify causes of delayed closure, and assess the rate of non-closure ileostomies along with potential risk factors. The secondary aim was to evaluate the morbidity and mortality associated with the ileostomy closure procedure and analyze the technical aspects used during surgery.

## Methods

This multicentre, prospective, observational study involved 45 high-volume colorectal surgery centres in Italy. Between February 15 and December 31, 2022, consecutive patients undergone diverting ileostomy following rectal cancer resection were prospectively enrolled at each centre. The inclusion criteria included patients over 18 years of age, histologically confirmed adenocarcinoma of the rectum, colorectal or coloanal anastomosis, and a protective ileostomy. Exclusion criteria included anterior rectal resection for conditions other than rectal cancer, other types of resections, prior surgery of the colon or rectum, or colostomies. Anonymized data were gathered using an online form, and at the conclusion of the study, the collected data were transferred to an electronic database. After the completion of patient enrollment, a minimum follow-up period of 6 months was implemented, with the dataset closing on June 30, 2023. The study received approval from the Institutional Ethics Committee (IEC) at the coordinating centre (Rovigo, Italy) as well as from the IECs of all the other centres. The research adhered to the ethical guidelines outlined in the 1964 Declaration of Helsinki and its later revisions. All patient details and associated variables were documented in the database. The recorded baseline characteristics included age, sex, body mass index (BMI), American Society of Anesthesiologists (ASA) score, hypertension status, and the location of rectal cancer. The cancer site was categorized based on its distance from the anorectal junction (ARJ): lower rectum (< 5 cm from the ARJ), middle rectum (5–9.99 cm from the ARJ), or upper rectum (10–15 cm from the ARJ), as determined by colonoscopy, digital rectal examination, and/or pelvic magnetic resonance imaging, as specified in the previous publication [8]. Methods for stoma creation variables have been accurately described in a previous manuscript, which focused on stoma creation [8]. Follow-up was defined as the period between stoma creation and the end of the study, which included a minimum follow-up period of 6 months, as per the study protocol. The stoma closure rate was defined as the ratio of the number of ileostomies closed during the follow-up period to the number of ileostomies created during the study period. The rate of stoma closure within 6 months was calculated and defined as the ratio between the number of stomas closed within 6 months of creation and the number of stomas created. The median closure time, defined as the number of days between the creation and closure of the stoma, was calculated. For each patient, the causes of non-stoma closure were acquired and reported in a database. Two features were evaluated in terms of surgical techniques for stoma closure: anastomotic techniques (stapled or hand-to-hand anastomosis) and skin-closure techniques (no closure, direct closure, or purse-string closure). During the post-operative period after stoma closure, the presence of short-term complications within 30 days of stoma closure was determined, and the rate of overall post-operative morbidity within 30 days was calculated. Moreover, the need for reoperation owing to post-operative complications and the occurrence of AL, post-operative small bowel obstruction, or skin leakage were recorded in the database. Univariate and multivariate tests were conducted to assess the factors affecting the stoma closure rate during the follow-up period, the 6-month stoma closure rate, and the 30-day post-operative complications. The variables included in the univariate and multivariate analyses to determine their impact on stoma closure outcomes were sex, age, ASA score, BMI, hypertension, cancer site, pre-operative stoma nurse and patient consultation, pre-operative stoma siting, stoma creation surgical procedure, alignment between the marked and actual stoma site, rod or baguette use, fixation of the stoma to the skin, occurrence of early and late post-operative stoma complications, availability of a stoma care nurse specialist, of a stoma service, and of a home care for patients with ostomy. Categorical variables were analyzed with chi-square or Fisher’s exact test, as appropriate. A significance threshold of *p* < 0.05 was applied. Significant variables or those with *p* < 0.125 in the univariate analysis were included in a multivariate logistic regression model to identify independent predictors of post-operative complications. All analyses were performed using the Wizard for MacOS software. The “STrengthening the Reporting of OBservational studies in Epidemiology” checklist was elaborated using formal items recommended for cohort studies from STROBE statement (https://www.strobe-statement.org/checklists/).

## Results

A total of 45 hospital centres in Italy participated in the study, with 287 patients (180 male; median age 71 years, range 33–88 years) included in the analysis. All patients underwent anterior resection with colorectal anastomosis and diverting ileostomy. The baseline patient characteristics are summarized in Table 1. Median BMI was 24.82 kg/m2, and 42.86% of patients had an ASA score greater than 2. Hypertension was present in 54.01% of patients. Extraperitoneal rectum was the most common cancer location, affecting 82.93% of patients. Factors influencing the decision to perform a diverting ileostomy included the distance of the colorectal anastomosis from the ARJ (58.19%), pre-operative chemoradiotherapy (49.13%), comorbidities (17.07%), and advanced age (9.41%). The results of stoma creation were reported in another article [8]. The median follow-up for all patients in the study was 9 months. By the end of the follow-up, 241 patients had their stoma closed, resulting in an overall stoma closure rate of 83.97%. Of these, 177 patients had their stoma closed within 6 months of creation, giving a 6-month stoma closure rate of 61.67%. The median time from stoma creation to closure was 146 days (range 9–483 days). The causes of the absence of stoma closure within the follow-up period in the 37 patients are reported in Table 2. The most frequent cause of lack of stoma closure was the necessity for adjuvant chemotherapy due to negative histopathological features. The most frequently used anastomotic technique was the hand-to-hand technique which was performed in 125 patients (52.08%). Twenty-one (8.75%) skin defects did not close in favour of second-intent closure. Approximately 70% of the skin defects were closed with direct sutures, while purse-string sutures were used in 47 patients (19.58%). In the multivariate analysis, factors independently related to stoma closure, ASA score > 2 (*p* = 0.028), advanced age (*p* = 0.048), and the occurrence of late stoma complications (*p* = 0.048) were found to be independently related to no stoma closure (Table 4). The overall short-term post-operative per-patient morbidity rate after stoma closure was 16.67% (40 patients). An anastomotic leak occurred in 5 patients (2.08%) and required reoperation in 2. Other complications were skin suture dehiscence in 17 patients (7.08%), requiring reoperation in one patient, and small bowel obstruction in 14 patients (5.83%), all treated conservatively (Table 3). In the multivariate analysis factors independently related to the occurrence of post-operative complications after stoma closure, only hypertension was found as an independent risk factor (*p* = 0.036) (Table 4).

**Table 1:** Baseline characteristics and stoma creation data

Variables	Data
*N. of patients enrolled*	287
*Sex (M:F)*	180 (62.72%):107 (37.28%)
*Median age (range)*	71 years (33–88 years)
*Median BMI (range)*	24.82 kg/m^2^ (15.8–46.7 kg/m^2^)
*ASA score*	I: 15 (5.23%)
	II: 149 (51.92%)
	III: 119 (41.46%)
	IV: 4 (1.39%)
*Hypertension*	155 (54.01%)
*Site of rectal cancer*	Upper rectum: 49 (17.07%)
	Mid rectum: 134 (46.69%)
	Low rectum: 104 (36.24%)
*Pre-operative consultation between patient and stoma nurse*	181 (63.07%)
*Marking of stoma site*	184 (64.11%)
*Laparoscopic approach for stoma creation*	231 (80.48%)
*Indications to perform ileostomy*	Distance between anastomosis and ARJ: 167 (58.19%)
	Pre-operative CRT: 141 (49.13%)
	Comorbidity: 49 (17.07%)
	Advanced age: 27 (9.41%)
	Positive anastomotic test: 12 (4.18%)
*Ratio between real and marked stoma site*	91.85%
*Use of rod*	171 (59.58%)
*Fixation of stoma to peristomal skin*	No: 10 (3.48%)
	Yes: 277 (96.52%)
*Mean postoperative length of hospital stay (range)*	7 days (3–92 days)

**Table 2:** Stoma closure data

Variables	Data
*Median follow-up (months)*	9 months (7–11 months)
*N. of stomas closed (stoma closure rate)*	241 (83.97%)
*N. of stomas closed within 6 months*	177 (61.67%)
*Median time for closure (range)*	146 days (9–483 days)
*Causes for no stoma closure*	Adjuvant chemotherapy: 13 (5.42%)
	Anastomotic leak: 7 (2.92%)
	Anastomotic stricture: 3 (1.25%)
	Organizational problems: 8 (3.33%)
	Death: 7 (2.92%)
	Comorbidity: 5 (2.08%)
	Progression of disease: 3 (1.25%)
*Anastomotic technique*	Mechanic: 115 (47.92%)
	Hand-to-hand: 125 (52.08%)
*Skin closure*	No closure: 21 (8.75%)
	Direct suture: 167 (69.58%)
	Purse-string: 47 (19.58%)
	Others: 5 (2.08%)

**Table 3:** Post-operative complications within 30 days from stoma closure

Variables	Data
*Overall post-operative complications*	40 (16.67%)
*Reoperation rate after stoma closure*	3 (1.25%)
*Anastomotic leak*	5 (2.08%)
*- Reoperations for anastomotic leak*	2
*Small bowel obstruction*	14 (5.83%)
*Skin suture dehiscence*	17 (7.08%)
*- Reoperation for skin suture dehiscence*	1

**Table 4:** Factors influencing stoma closure rate and post-operative complications after stoma closure

Outcome	Univariate analysis (*p*-value)	Multivariate analysis (*p*-value)
*Stoma closure*	Age (0.072) Sex (0.474) ASA > 2 (0.086) Respect of pre-operative marked stoma site (0.073)	ASA > 2 (*p*: 0.028)
*Stoma closure within 6 months*	Age (0.040)* (better younger) Sex (0.031)* (better females) Use of rod (0.028)* (better in rod used) Stoma retraction (0.081) Other late stoma-related complications (0.018)* (better in no complications)	Sex (0.048) Other late stoma related complications (0.048)
*Post-operative complications after stoma closure*	Hypertension (0.041) Pre-op consultation (0.062)	Hypertension (0.036)

## Discussion

This article is part of a study on the role of diverting ileostomies following rectal cancer resection conducted in 45 Italian surgical centres that perform colorectal surgery. A previous study analyzed the outcomes of ileostomy creation [8]. The present study revealed that the stoma closure rate was approximately 84%, with a 6-month closure rate of approximately 62% and a median stoma closure time of 146 days. The most frequent cause of non-stoma closure is adjuvant chemotherapy. Technical aspects of the ileostomy closure procedure were also investigated: hand-to-hand anastomosis was realized in approximately 52% of patients, approximately 70% of skin defects were closed with direct suture, while a purse-string suture was used in only 20% of cases. The overall short-term morbidity rate was approximately 17%, requiring reoperation in only three cases. Protective loop ileostomy is usually performed in patients with middle and lower rectal cancers, especially in cases of high-risk colorectal or coloanal anastomoses. In our previous study, we found that the primary reasons for performing a protective ileostomy were the distance of the anastomosis from the ARJ (58%), pre-operative chemoradiotherapy (49%), concomitant comorbidities (17%), and advanced age (9%) [8]. Ileostomy is presumed to prevent or reduce the potentially catastrophic consequences of AL, with rectal cancer surgery being the most common complication [15]. Several studies have demonstrated a reduction in the AL rate of approximately 50% in patients with diverting ileostomy [15]; however, this procedure is not free from complications, not only in the post-operative phase, but also after its closure [16]. One of the most important issues regarding diverting ileostomies is the closure timing, which is the principal aim of this study. The optimal timing for ileostomy reversal remains a subject of ongoing debate, as it must balance the risks of early closure with complications associated with prolonged stoma maintenance. In our study, the median time to closure was recorded as 146 days, with a range of 9 to 483 days, and with a closure rate of 83.97% in the entire population during the 9-month follow-up (range 7–11 months) and a 6-month closure rate of 61.67%. Traditionally, ileostomy closure is often performed several months after the initial surgery, once the anastomosis has healed and any complications have been addressed. However, recent evidence has prompted a re-evaluation of this timing, with some advocating earlier closure to reduce stoma-related complications and improve the patient’s quality of life [17, 18]. Early closure can mitigate the physical and psychological burdens associated with stomas, such as skin irritation, dehydration, and social discomfort. Moreover, the early restoration of bowel continuity is associated with a lower risk of stoma-related complications, including parastomal hernias and high-output stomas, which can lead to significant morbidity. On the other hand, late closure allows ample time for the anastomosis to heal and for any post-operative complications to be managed effectively. Moreover, it may also be advantageous for patients undergoing adjuvant chemotherapy, as it allows the primary treatment to proceed without the added risk of complications from ileostomy closure. Guidelines recommend that early closure may be considered feasible and safe in patients who experience an uneventful recovery and show no signs of AL [14, 19]. In the literature, conventional closure timing, defined as 8–12 weeks or more after surgery, has been compared to early timing, defined as 2–4 weeks after surgery. Several studies have shown no significant differences in morbidity, reoperation rates, or leakage of the primary anastomosis between early and late closure in patients undergoing ileostomy closure within 30 days post-surgery [20–23]. However, some researchers have noted a higher incidence of wound complications in patients undergoing early closure [23, 24]. A systematic review and meta-analysis by O’Sullivan et al. compared 275 patients who had early stoma closure with 259 patients who had standard closure. The study concluded that early closure appears a viable option for carefully selected patients with appropriate perioperative counseling and shared decision-making. However, they observed a higher rate of reoperations (8.4% vs. 4.2%) and small bowel obstructions/postoperative ileus (9.3% vs. 4.4%) in the early closure group compared to those who had late closure [25]. Our study registered delayed stoma closure times (median 146 days, > 20 weeks), which is very far not only from the early closure time of 2–4 weeks but also from the late closure time of 8–12 weeks described and proposed in the literature. We also investigated the stoma closure rate within the follow-up period of 9 months, which was > 80%, and focused on the remaining 16.03% of patients who had their ileostomies not closed during this period. Moreover, more patients had their ileostomy not closed before 6 months (38.33%). In fact, there is a huge variation in time-to-closure periods across Europe. In some countries, such as Scandinavian countries, a closure time > 3 months after the initial surgery is considered late [26]; instead, 34% of ileostomies after rectal resection remain unclosed at 18 months, in the UK [27]. Therefore, the results of our study may be considered consistent with the general trends registered across Europe, even if greater efforts are required to improve these outcomes. We also investigated the possible causes of delayed or missed closure; the most frequent cause of no stoma closure was the necessity of adjuvant chemotherapy, followed by organizational issues and anastomotic problems (Table 2). Ileostomy closure is usually realized after adjuvant chemotherapy, which may explain the delay in or missed stoma closure in some patients. However, some studies have demonstrated no difference in post-operative complications, specifically AL, surgical site infection, and ileus, in patients who had loop ileostomy closure either during or following adjuvant chemotherapy [28–30]. Risk factors for stoma closure were also investigated, demonstrating that higher ASA, advanced age, and the occurrence of late stoma complications may be associated with delay or no stoma closure. The CLOSE-IT study from the UK, which registered a median time to closure of 259 days, reported AL, chemotherapy, and cancer progression as factors associated with the delay and risk of no closure [31].

Our study reported a complication rate of approximately 17% after loop ileostomy reversal, which is in line with that reported in the literature, in which complication rates can reach 20% [32, 33] and necessitate reoperation in 12–15% of cases [34, 35]. In a large cohort of patients (from the NSQIP database) who underwent ileostomy closure, 9.3% had major complications and 8.4% had minor complications, with a mortality rate of 0.6% [16]. Our study registered a low reoperation rate (only three patients, 1.25%), of which two patients underwent reoperation for AL and one for skin closure problems. The overall AL rate was 2.08% (5 patients), and only 2 patients required reoperation. Climent et al. reported an AL rate of 2.3% (10 patients) after ileostomy reversal, and five patients underwent reoperation [36]. The overall rate of AL reported by Vergara-Fernández et al. was 5.1%; however, they included patients with both benign and malignant diseases [37]. Other complications registered in our study were small bowel obstruction (SBO), with an incidence of 5.83%, and skin leakage, with a rate of 7.08% (seven patients, one requiring reoperation). The first is commonly reported in other studies, with an incidence of 5.1–28.7%, sometimes requiring reoperation; however, in our study, none of the patients with SBO underwent reoperation [38, 39]. Although SBO is generally regarded as a minor complication, it can impact the post-operative recovery by extending hospital stays or necessitating parenteral nutrition in certain patients, which brings its own set of associated morbidities. Unfortunately, none of these aspects were analyzed in detail in our study, especially those related to specific complications such as SBO. The relatively low incidence of SBO in our series may be attributed to the high proportion of laparoscopic surgeries performed (80.48% [8]), which can be linked to a reduced risk of SBO. Laparoscopy in fact minimizes surgical trauma, abdominal wall incisions, and tissue manipulation, leading to fewer adhesions [40]. The factors influencing post-operative complications after stoma closure were also analyzed. The presence of comorbidities, including diabetes, cardiovascular disease, and chronic kidney disease, is strongly associated with higher rates of post-operative complications. Cardiovascular diseases can exacerbate surgical stress and increase the likelihood of cardiopulmonary complications, further complicating recovery [36, 37, 41]. In our study, only hypertension was identified as a potentially significant risk factor in both univariate and multivariate analyses. Lv et al. reported a correlation between pre-operative hypertension and stoma-related complications after ileostomy reversal [42]. The relationship between hypertension and complications following ileostomy closure has not been extensively studied in the literature; however, some indirect evidence suggests that hypertension might contribute to an increased risk of post-operative complications, although it may not be a direct cause. Hypertension is a significant risk factor for cardiovascular diseases that can complicate the perioperative period. Patients with hypertension are more likely to experience cardiovascular complications such as myocardial infarction, stroke, or arrhythmias, particularly during or after surgery. These complications can adversely affect the overall outcomes of ileostomy closure [43]. Moreover, hypertension can contribute to impaired wound healing because of its effects on the blood vessels. Chronic hypertension may lead to changes in microcirculation, reduce blood flow to surgical sites, and potentially increase the risk of wound infections and poor anastomotic healing. These issues, which are not unique to ileostomy closure, could be more pronounced in patients with hypertension, leading to higher rates of ALs or post-operative infections [44]. Finally, hypertension often coexists with other comorbidities, such as diabetes, obesity, and chronic kidney disease, all of which are known to increase the risk of post-operative complications. Therefore, while hypertension alone may not be a direct risk factor, its presence, in conjunction with other conditions, can elevate the overall risk profile of patients undergoing ileostomy closure [45, 46].

Some technical aspects may be related to the occurrence of post-closure complications. In our study, the anastomotic technique and skin closure were investigated. The choice of anastomotic technique is influenced by factors such as surgeon’s preference, patient anatomy, and clinical context. Some studies suggest that hand-sewn anastomosis is associated with comparable outcomes to stapled anastomosis, particularly in terms of AL rates; however, the stapled technique is considered superior to hand-sewn closure in terms of lower early post-operative small-bowel obstruction rate and shorter operative time [14, 47]. In our study, the two techniques were almost equally distributed in the population (stapled 47.92% vs. hand-to-hand 52.08%), and no correlation was observed with the post-operative complications or stoma closure rate.

The choice of skin closure technique after ileostomy closure is known to influence the post-operative surgical site infection rate. This study did not find any correlation between the skin closure technique and post-closure complications; however, we have to highlight the relatively low rate of purse-string skin closure compared with direct suturing (19.58% vs. 69.58%, respectively). This contrasts with recent evidence and guidelines that suggest purse-string closure as the preferred method for stoma reversal due to its association with lower rates of surgical site infections compared to other techniques [14, 19]. Additionally, purse-string closure may offer other benefits over linear closure, including improved patient satisfaction and a reduced incidence of complications like incisional hernias. A recent Cochrane systematic review concluded that the purse-string suture technique has several advantages over traditional linear closure and should be recommended as a standardized skin closure method for stoma reversal [48].

This study had some limitations. This was a multicentre study involving different Italian surgical centres, which may not be representative of all countries. Moreover, there is a limited description of the causes related to the delay in stoma closure or lack of stoma closure; moreover, we are not aware of the clinical outcomes after the study inclusion period, especially in patients with no stoma closure. Finally, some risk factors and complications for each patient were not collected or analyzed.

In conclusion, this study reported that in Italy, a significant delay exists in ileostomy reversal timing after rectal cancer resection, and a high number of ileostomies do not close within 6 months. Approximately 16% of ileostomies are still not closed after 9 months, with reasonable doubt as to whether these patients should be considered to have a permanent stoma. Post-operative complications are still high; however, in line with the literature, they can be prevented by adequate pre-operative assessment and post-operative care. The most common skin closure technique is a linear closure; however, it needs to be changed in favour of a purse-string suture, which is recognized as the best method to reduce post-operative complications and improve patient satisfaction.

## Data Availability

The data that support the findings of this study are available from the corresponding author, upon reasonable request.
